# Altered brain connectivity in hyperkinetic movement disorders: A review of resting-state fMRI

**DOI:** 10.1016/j.nicl.2022.103302

**Published:** 2022-12-24

**Authors:** Ramesh S. Marapin, Harm J. van der Horn, A.M. Madelein van der Stouwe, Jelle R. Dalenberg, Bauke M. de Jong, Marina A.J. Tijssen

**Affiliations:** a University Medical Center Groningen, Hanzeplein 1, 9713 GZ Groningen, the Netherlands; b Expertise Center Movement Disorders Groningen, University Medical Center Groningen (UMCG), Groningen, the Netherlands

**Keywords:** Hyperkinetic movement disorders, Resting state fMRI, Brain networks, Pathophysiology, Review

## Abstract

**Background:**

Hyperkinetic movement disorders (HMD) manifest as abnormal and uncontrollable movements. Despite reported involvement of several neural circuits, exact connectivity profiles remain elusive.

**Objectives:**

Providing a comprehensive literature review of resting-state brain connectivity alterations using resting-state fMRI (rs-fMRI). We additionally discuss alterations from the perspective of brain networks, as well as correlations between connectivity and clinical measures.

**Methods:**

A systematic review was performed according to PRISMA guidelines and searching PubMed until October 2022. Rs-fMRI studies addressing ataxia, chorea, dystonia, myoclonus, tics, tremor, and functional movement disorders (FMD) were included. The standardized mean difference was used to summarize findings per region in the Automated Anatomical Labeling atlas for each phenotype. Furthermore, the activation likelihood estimation meta-analytic method was used to analyze convergence of significant between-group differences per phenotype. Finally, we conducted hierarchical cluster analysis to provide additional insights into commonalities and differences across HMD phenotypes.

**Results:**

Most articles concerned tremor (51), followed by dystonia (46), tics (19), chorea (12), myoclonus (11), FMD (11), and ataxia (8). Altered resting-state connectivity was found in several brain regions: in ataxia mainly cerebellar areas; for chorea, the caudate nucleus; for dystonia, sensorimotor and basal ganglia regions; for myoclonus, the thalamus and cingulate cortex; in tics, the basal ganglia, cerebellum, insula, and frontal cortex; for tremor, the cerebello-thalamo-cortical circuit; finally, in FMD, frontal, parietal, and cerebellar regions. Both decreased and increased connectivity were found for all HMD. Significant spatial convergence was found for dystonia, FMD, myoclonus, and tremor. Correlations between clinical measures and resting-state connectivity were frequently described.

**Conclusion:**

Key brain regions contributing to functional connectivity changes across HMD often overlap. Possible increases and decreases of functional connections of a specific region emphasize that HMD should be viewed as a network disorder. Despite the complex interplay of physiological and methodological factors, this review serves to gain insight in brain connectivity profiles across HMD phenotypes.

## Highlights

• Hyperkinetic movement disorders (HMD) should be viewed as network disorders.
• Resting-state brain connectivity changes often overlap across HMD.
• Besides overlap, distinct brain changes spatially discriminate between HMD.
• For many HMD, significant convergence in brain regions remains unidentified.

## 1 Introduction

Hyperkinetic movement disorders (HMD) are neurological syndromes with an excess of abnormal and uncontrollable movements, comprising six main phenotypic categories: chorea, dystonia, myoclonus, tics, tremor, and stereotypies. (Albanese and Jankovic, 2012, Fahn, 2011) Cerebellar ataxia may share overlapping clinical features with these phenotypes, making it relevant to include in this review. The pathophysiology of HMD remains largely unclear. Currently, neurophysiological and neuropathological evidence points to the cerebello-thalamo-cortical (CTC) circuit in tremor and myoclonus. (Helmich et al., 2013, Pinto et al., 2003, van der Stouwe et al., 2020, Hallett, 2014, Buijink et al., 2016, Buijink et al., 2015, Latorre et al., 2020, Ganos et al., 2014, Tijssen et al., 2000) In dystonia, basal ganglia and CTC pathways are implicated. (Vidailhet et al., 2009, Vidailhet et al., 2012, Hallett, 2006) For tics and chorea, evidence points to the basal ganglia-thalamo-cortical (BGTC) circuit. (Berardelli et al., 1999, Berardelli et al., 2003, Albin, 1995) Cerebellar ataxia may result from damage to the cerebellum, as well as cerebellar pathways. (Marsden and Harris, 2011, Manto and Marmolino, 2009, Stefanescu et al., 2015) While several regions are implicated, the emerging concept is that dysfunction arises at the level of cerebral networks in these disorders, sometimes showing spatial overlap.

To explore connectivity profiles of specific phenotypes in HMD, resting-state fMRI (rs-fMRI) can be employed, which measures spontaneous low-frequency fluctuations in the blood-oxygen-level-dependent (BOLD) signal. (Lee et al., 2013) In this context, highly synchronized brain regions correspond to functionally relevant intrinsic resting-state networks. (Lee et al., 2013, Biswal et al., 1995, Damoiseaux et al., 2006) As our brain is considered a network of interconnected regions, rs-fMRI can potentially advance our understanding of the pathophysiology underlying HMD.

Functional connectivity (FC) analysis identifies the level of coactivation (synchrony) of the BOLD time-series between different brain regions. It provides information about functional connections and not about a *specific* region per se. Graph theory methods model the brain as a network represented by a collection of “nodes” (brain regions) and “edges” (*connections* between nodes). (Wang et al., 2010) In contrast to FC, graph-based network analyses allow us not only to describe the overall connectivity pattern among brain regions but also to quantitatively characterize the global organization (e.g., network efficiency). Two other analysis methods that are commonly used include measuring amplitude of low frequency fluctuations (ALFF) and regional homogeneity (ReHo) of *specific brain regions.* ALFF measures the total signal power (square of the amplitude) in the low-frequency range and can be computed for every voxel or region of interest. ReHo analyzes a specific voxel and measures its relationship with nearby voxels. (Canario et al., 2021) While ALFF and ReHo have been suggested to reflect spontaneous neuronal activity, their physiological relevance remains disputed. (Canario et al., 2021, Zang et al., 2007) As several analysis methods are used to investigate resting-state signal fluctuations and synchronization in rs-fMRI, differences between methods as well as the functional relevance need to be taken into account. In this respect, it should be noticed that disease-related neuronal loss in distinct brain regions generally results in a reduced diversity of structural connections within a network while, on the other hand, residual connections may yield relatively increased FC between some of the interconnected regions. Table 1 provides a short description of commonly used analysis methods.

**Table 1:** Definitions of rs-fMRI analysis methods used in this systematic review.

Analysis method	Definition
**Functional connectivity (FC)**	FC is defined as statistical dependencies among remote neurophysiological events. (Friston, 2011)
FC density (FCD)	Measure of the number of connections between a given voxel and all the other remaining brain voxels. (Tomasi and Volkow, 2010)
Voxel-mirrored homotopic connectivity (VMHC)	Measure of FC between each voxel and its symmetrical interhemispheric counterpart (voxel). (Zuo et al., 2010)
Seed-based FC (also called region of interest (ROI)-based FC)	Measure of FC between seed region(s) and the rest of the brain. (Lv et al., 2018)
Independent component analysis (ICA)	Multivariate data decomposition of spatially distributed BOLD signals into independent, temporally correlated functional components (networks) expressed as spatial maps with their corresponding time-series. (Beckmann et al., 2005) Several resting-state networks emerge from ICA analysis, e.g., the default mode network. (Damoiseaux et al., 2006)
Stepwise FC (SFC)	Measure of FC that characterizes the propagation and convergence of FC across brain networks. (Sepulcre et al., 2012)
**Graph analysis**	Analysis of brain networks by examining both the local and global connectivity of networks. (Lv et al., 2018, Rubinov and Sporns, 2010)
Clustering coefficient	Describes the connectedness of the direct neighbors of the nodes and gives information on the formation of sub-graphs within the full network. (van den Heuvel et al., 2008)
Characteristic path length	Describes the average shortest path length between all pairs of nodes in the network and gives information on the global level of connectedness of a network. (van den Heuvel et al., 2008)
Degree	Describes the number of connections of a node, helps identify highly connected nodes within a network. (Rubinov and Sporns, 2010)
Efficiency	Global efficiency and local efficiency measure the network ability to transmit information at the global and local level, respectively. (Wang et al., 2010)
Modularity	Describes the extent to which groups of nodes are connected to members of their own group and reflects the existence of subnetworks within the full network. (Lv et al., 2018)
**Amplitude of low frequency fluctuations (ALFF)**	Measure of the total power of BOLD signal within the low-frequency range of 0.01–0.08 Hz and is proportional to regional neural activity. (Zang et al., 2007)
Fractional ALFF (fALFF)	ALFF-variant that measures power within the low-frequency range divided by total power in the entire detectable frequency range. (Zou et al., 2008)
**Regional Homogeneity (ReHo)**	Measure of similarity between BOLD time-series of a voxel and its nearest neighbors, calculated by Kendall coefficient of concordance of the time-series. (Lv et al., 2018, Zang et al., 2004)

To date, several rs-fMRI studies have been performed in HMD, employing many different methods. Our aim is to give a comprehensive review of the affected brain regions in HMD and put them into context with each other. We therefore performed a systematic review and meta-analysis to investigate HMD findings from rs-fMRI studies, provide an overview of the brain connectivity alterations, and discuss the pathophysiology of several HMD, including chorea, dystonia, myoclonus, tics, tremor, and we also reviewed ataxia and functional movement disorders (FMD). The overarching term “connectivity” is used to describe changes in resting-state signal fluctuations and synchronization found using the previously described analysis methods. In addition, to gain further insight into the role of involved brain regions, we will discuss the relationship between altered resting-state connectivity and clinical characteristics.

## 2 Methods

### 2.1 Search strategy

A systematic literature search was performed according to the PRISMA guidelines. (Moher et al., 2009) We included studies on chorea, dystonia, myoclonus, tics, tremor (including tremor-dominant Parkinson’s disease [PD]), ataxia, and FMD. Non-tremor-dominant PD was excluded. We searched the literature via PubMed until October 2022. A combination of several free text and MeSH terms were used for the literature search, mainly including “MRI”, “Movement Disorders”, “ataxia”, “chorea”, “dystonia”, “functional neurological disorder”, “myoclonus”, “tics”, “tremor”, and “rest”. Additionally, we performed a targeted search using articles’ reference lists to identify additional studies of interest. The complete details regarding the search strategy and extracted information is further elaborated in the Supplementary material.

### 2.2 Screening procedure

Three of the authors (RM, AMMS, and HJvdH) independently reviewed the titles and abstracts, blinded to authors and journal titles, using an Excel workbook designed specifically for screening. (VonVille, 2021) Items were reviewed in pairs, and in cases where there was disagreement, cases were discussed by the two article reviewers until consensus was reached. If consensus could not be reached, the third reviewer provided final arbitration. We included studies if they reported rs-fMRI findings in patients with the HMD and FMD described above.

### 2.3 Reporting of results

Results per phenotype will be discussed in three subparagraphs: 1) changes in resting-state signal fluctuations and synchronization, which entails differences in connectivity between patients and healthy participants; 2) changes in resting-state networks, where we discuss studies that investigated resting-state networks; 3) correlation between resting-state connectivity and clinical measures. Due to the vastness of results, we mostly report correlations between brain function and motor severity, except for myoclonus and FMD where other clinical measures are also described. As articles used different brain atlases and terminology to label brain regions, we transformed the regions to a standardized template based on the automated anatomical labeling (AAL) atlas to unify neuroanatomical nomenclature. (Tzourio-Mazoyer et al., 2002).

**Fig. 1:**
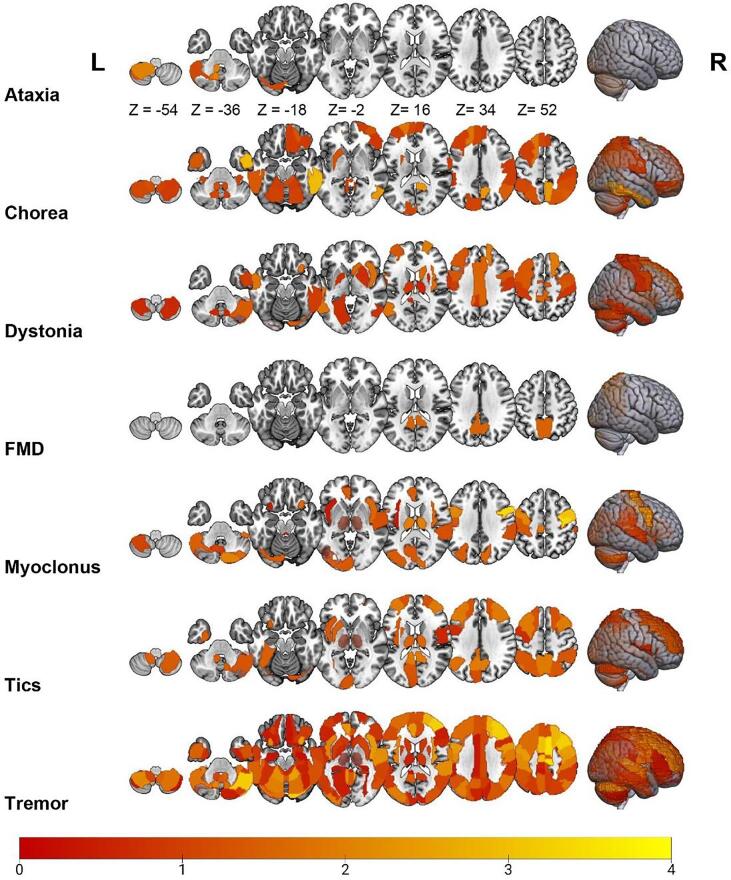
Overview of resting-state fMRI alterations found using ALFF and ReHo analysis methods across several HMD. The color of a particular brain region reflects the degree of involvement according to the standardized mean difference of each brain region. The brighter the color (i.e., from red to yellow), the more involved that particular brain region is for that movement disorder. (For interpretation of the references to color in this figure legend, the reader is referred to the web version of this article.)

**Fig. 2:**
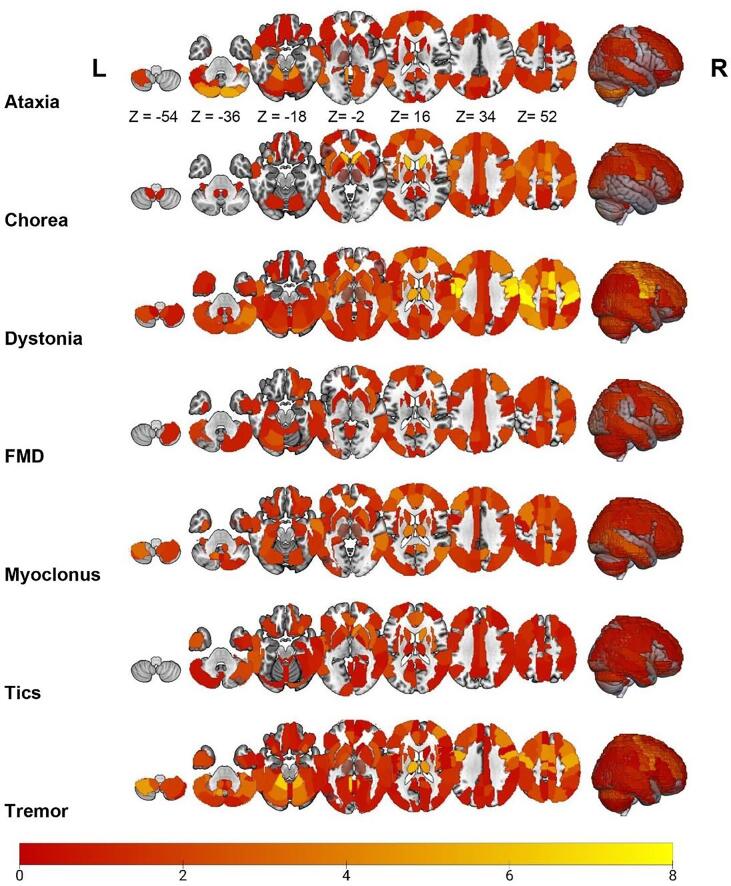
Overview of resting-state fMRI alterations found using FC and graph analysis methods across several HMD. The color of a particular brain region reflects the degree of involvement according to the standardized mean difference of each brain region. The brighter the color (i.e., yellow), the more involved that particular brain region is for that movement disorder. (For interpretation of the references to color in this figure legend, the reader is referred to the web version of this article.)

Here, we briefly describe how we report our results. We calculated a standardized mean difference (SMD) for each study result when possible, which was done for the changes in resting-state signal fluctuations and synchronization. The SMD was calculated using Wilson’s Practical meta-Analysis Effect Size Calculator. (Ialongo, 2016, Nuyts et al., 2017, Thomas et al., 2020, Wilson) To summarize findings per HMD phenotype, we first calculated the absolute value (i.e., only positive values) of the SMD, in order to reflect the degree of involvement of each region per HMD phenotype (Fig. 1, Fig. 2). Furthermore, we constructed HMD brain signature maps by averaging SMDs across studies and AAL brain regions, to describe the overall direction (i.e., positive or negative) of our findings (Supp. Figs. 2–3). It should be noted that several brain regions showed results in opposite directions, i.e., both increased and decreased connectivity within the same HMD phenotype. Therefore, caution is warranted when interpreting these results. We visualized the changes in connectivity by weighing each brain region in the AAL atlas according to the degree of involvement (Fig. 1, Fig. 2) and direction of connectivity (Supp. Figs. 2–3) and overlaying this map on a volume rendered brain (using Chris Rorden’s MRIcroGL version 1.2.20220720.). A custom made MATLAB (version 2020a) script was used to create these maps. Functional connectivity and graph analysis assesses the brain as an integrated network and focuses on the connectivity between different brain areas, whereas ALFF and ReHo analyses focus on regional brain connectivity. (Lv et al., 2018) As these methods measure different properties of brain functioning, we visualize the results of these methods separately.

**Fig. 3:**
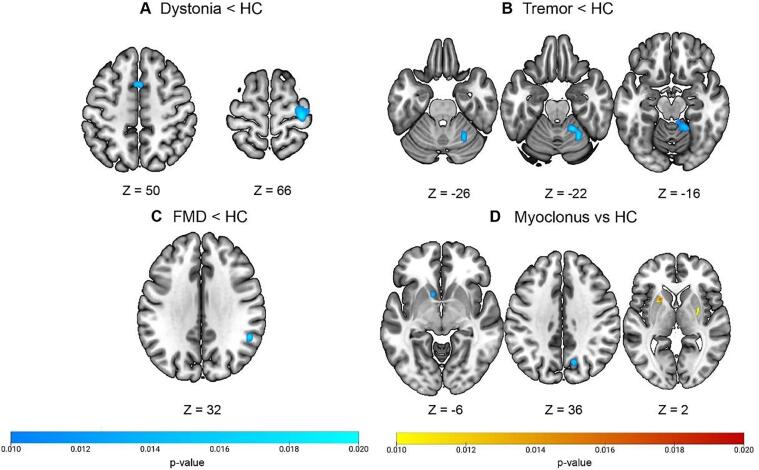
Activation likelihood estimation (ALE) maps for the (A) dystonia < HC contrast, (B) tremor < HC contrast, (C) FMD < HC contrast, and (D) myoclonus vs HC contrasts. Brain maps show significant brain clusters exhibiting convergence, where blue and yellow–red signify hypo- and hyperconnectivity, respectively (p ≤ 0.001, uncorrected at the voxel-level, p ≤ 0.05, FWE-corrected at the cluster-level). ALE maps were computed using GingerALE (version 3.0.2). (For interpretation of the references to color in this figure legend, the reader is referred to the web version of this article.)

Furthermore, in order to provide a quantitative synthesis of the rs-fMRI studies across phenotypes, we additionally conducted a meta-analysis using the activation likelihood estimation (ALE) approach. (Eickhoff et al., 2012) This approach evaluates brain regions in which the convergence of reported hyperconnectivity or hypoconnectivity across studies is higher than would be expected by chance. Therefore, the main question that is addressed is: where in the brain have hyperconnectivity or hypoconnectivity of brain regions in a particular disorder consistently been reported across studies? (Cortese et al., 2021) We determined convergence of significant between-group differences using GingerALE (version 3.0.2; https://www.brainmap.org/ale/). We did this for each phenotype, making separate maps for hyperconnectivity (patients > healthy controls) and hypoconnectivity (healthy controls > patients). For each phenotype, we only included studies that reported significant results as coordinates in standard space. Results that were not reported in coordinates were not considered for the meta-analysis. Significant convergence (at p < 0.001, uncorrected at the voxel-level, p < 0.05, FWE-corrected at the cluster-level) for phenotypes is shown in Fig. 3, whereas unthresholded (positive) z-score maps of the ALE analysis are shown in Supplementary Figure 4.

**Fig. 4:**
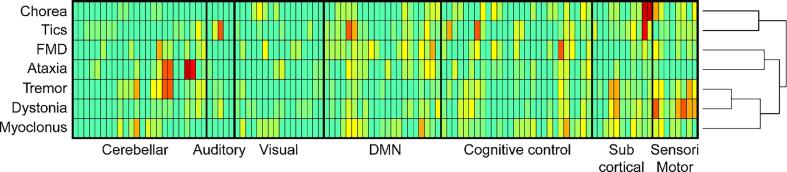
Commonalities and differences in implicated brain regions across HMD phenotypes. The columns of the heat map represent different HMD phenotypes and the rows represent functional brain networks. For visibility purposes, horizontally, we have grouped the AAL brain regions used in this study into larger scale networks, similarly to the work by Allen et al. (Allen et al., 2014) The effect sizes of the implicated brain regions are transformed into colours, where regions (rows) that are more red are more involved in the phenotype (column). The figure therefore shows which brain regions (networks) are commonly and differentially involved across different HMD. Phenotypes which show alterations in similar brain networks can be easily seen using this heat map, as they are grouped closer together. For our HCA, we used the agglomerative strategy, which is a “bottom up” approach. Initially, both brain regions and HMD are assigned to their own clusters, and the algorithm proceeds iteratively, at each stage joining the two most similar cluster. This process continues until there is a single cluster. The heat map was made using R Statistical Software (version 4.0.0; R Core Team 2020) with the *heatmap.2* function in the gplots package: https://cran.r project.org/web/packages/gplots/. (Warnes et al., 2020). (For interpretation of the references to color in this figure legend, the reader is referred to the web version of this article.)

Finally, we also conducted hierarchical cluster analysis (HCA) to provide additional insights into commonalities and differences across HMD phenotypes (Fig. 4). (Zhang et al., 2017) Essentially, HCA attempts to group objects (i.e., HMD phenotypes) with similar features into clusters. For visibility purposes, horizontally, we grouped the AAL brain regions used in this study into domains, similarly to the work by Allen et al. (Allen et al., 2014) The effect sizes of the implicated brain regions are transformed into colors, where regions (rows) that are more red are more involved in the phenotype (column), forming a heat map. Such a figure shows which brain regions (networks) are commonly and differentially involved across different HMD. Phenotypes which show alterations in similar brain networks can be easily seen using this heat map, as they are grouped closer together. For our HCA, we used the agglomerative strategy, which is a “bottom up” approach. Initially, both brain regions and HMD are assigned to their own clusters, and the algorithm proceeds iteratively, at each stage joining the two most similar cluster. This process continues until there is a single cluster. The heat map was made using R Statistical Software (version 4.0.0; R Core Team 2020) with the *heatmap.2* function in the gplots package: https://cran.r project.org/web/packages/gplots/. (Warnes et al., 2020) The complete details on how we report and visualize our findings can be found in the Supplementary material.

The datasets generated during and/or analyzed during the current study are available from the corresponding author upon reasonable request.

## 3 Results

### 3.1 Search results

Our search resulted in 1052 articles, of which 29 were duplicates and 818 articles were rejected based on titles and abstracts (Supplementary Fig. 1). Next, 205 articles were reviewed, of which 148 studies were included in the final analysis. In addition, 10 articles were added from references of the included articles. Exclusion criteria were: 1) wrong patient group (e.g., schizophrenia [n = 602]); 2) study without rs-fMRI (n = 125); 3) review or case report (n = 73); 4) analysis not of interest (n = 52); 5) sample size too small (n = 14); 6) dataset previously examined in the included articles using similar analysis methods (n = 9). Taken together, a total of 158 papers were identified, concerning the phenotypes ataxia (n = 8), chorea (n = 12), dystonia (n = 46), FMD (n = 11), myoclonus (n = 11), tics (n = 19), and tremor (n = 51).

### 3.2 Ataxia

Eight articles on ataxia patients were included in this review. (Jiang et al., 2019, Ren et al., 2019, Cocozza et al., 2015, Cocozza et al., 2018, Pereira et al., 2017, Hernandez-Castillo et al., 2013, Hernandez-Castillo et al., 2015, Chen et al., 2022) Patients were diagnosed with spinocerebellar ataxia type 2 (SCA2), SCA3, SCA7, SCA6, sporadic adult-onset of ataxia, multiple system atrophy with cerebellar atrophy, and Friedreich’s ataxia. All studies compared patient groups with age- and gender-matched healthy participants. The number of patients per study varied from 12 to 41.

#### 3.2.1 Changes in resting-state connectivity

Most studies found differences between patients and healthy participants, except for one study conducted in 37 sporadic adult-onset of ataxia patients. (Jiang et al., 2019) Most frequently, three studies found decreased FC in the left cerebellum lobule IV-V. (Ren et al., 2019, Hernandez-Castillo et al., 2013, Hernandez-Castillo et al., 2015) Other regions in the cerebellum, frontal, parietal, and temporal cortex were also implicated (Fig. 1, Fig. 2, Supp. Figs. 2–3). The effect in several of these regions was highly variable, i.e., exhibiting both increased and decreased connectivity. The ALE analysis (two and four studies for the hyper- and hypoconnectivity contrasts, respectively) did not show any statistically significant clusters in both contrasts.

#### 3.2.2 Changes in resting-state networks

Increased global efficiency was shown in the visual networks of patients with SCA6, using graph-based network analysis. (Pereira et al., 2017) Another network study found increased FC in a default mode (DMN) and cognitive control network (CCN), and decreased FC in a cerebellar network in patients with SCA2. (Hernandez-Castillo et al., 2015) In contrast, decreased FC has also been reported in DMN and CCN in SCA2 patients. Although opposite effects may be due to different loci these networks are interconnected with, it more likely reflects inconsistency of results. (Cocozza et al., 2015).

#### 3.2.3 Correlation with clinical variables

Two of seven studies found no correlation between clinical measures and FC. (Cocozza et al., 2015, Cocozza et al., 2018) Two studies describe positive relationships between motor severity and parameters of brain function: 1) global efficiency in a visual network; 2) FC between the right cerebellum crus 1 and right frontal cortex. (Pereira et al., 2017, Hernandez-Castillo et al., 2015) Three studies found negative relationships between motor severity and brain function: 1) FC between left cerebellum lobule VIII and the left parahippocampal gyrus, inferior parietal lobule, and right lingual gyrus; 2) global efficiency in a CCN; 3) FC in a cerebellar network. (Ren et al., 2019, Pereira et al., 2017, Hernandez-Castillo et al., 2015) Two studies found a negative correlation between disease duration and FC: between the right cerebellum crus 1 and left frontal cortex, and between bilateral calcarine cortex and right caudate nucleus. (Hernandez-Castillo et al., 2013, Chen et al., 2022) The latter study also described a positive correlation between disease duration and FC between the posterior cingulate cortex and the occipital gyrus and cerebellum. One study found a negative correlation between the number of CAG triplets in SCA7 and FC between the right cerebellum crus 1 and the left frontal and parahippocampal gyrus. (Hernandez-Castillo et al., 2013).

### 3.3 Chorea

For chorea, 12 articles were included, (Sanchez-Castaneda et al., 2017, Sarappa et al., 2017, Dumas et al., 2013, Aracil-Bolaños et al., 2022, Gargouri et al., 2016, Liu et al., 2016, Muller et al., 2016, Wolf et al., 2015, Wolf et al., 2014, Werner et al., 2014, Quarantelli et al., 2013, Poudel et al., 2014) all concerning Huntington’s disease (HD) and well-matched for age and gender. The number of patients per study varied from 10 to 43.

#### 3.3.1 Changes in resting-state connectivity

Most studies found differences between patients and healthy participants, except for one study conducted in 20 HD patients. (Wolf et al., 2015) The caudate nucleus was most frequently implicated (n = 5), showing both increased and decreased connectivity. (Aracil-Bolaños et al., 2022, Muller et al., 2016, Wolf et al., 2014, Werner et al., 2014, Quarantelli et al., 2013) Among other regions, the superior frontal, parietal, and inferior temporal cortex similarly showed both increased and decreased connectivity (Fig. 1, Fig. 2, Supp. Figs. 2–3). The ALE analysis (four and seven studies for the hyper- and hypoconnectivity contrasts, respectively) did not show any statistically significant clusters in both contrasts.

#### 3.3.2 Changes in resting-state networks

One study found HD-related FC increases in the DMN and sensorimotor network (SMN), (Sanchez-Castaneda et al., 2017) while another reported reduced clustering in the SMN together with increased degree in associative and limbic networks. (Gargouri et al., 2016) In addition, one study described decreased FC in the DMN and salience network (SN). (Aracil-Bolaños et al., 2022) Decreased connectivity between parieto-occipital networks, which partly coincide with the DMN, has also been described, (Werner et al., 2014) fuelling an apparent inconsistency of results for the DMN and SMN in patients with chorea. Another study found decreased FC in a CCN. (Poudel et al., 2014).

#### 3.3.3 Correlation with clinical variables

Three of 10 studies found no significant correlation between clinical measures and resting-state brain connectivity. (Sarappa et al., 2017, Liu et al., 2016, Quarantelli et al., 2013) Two studies described positive relationships between motor severity and brain function parameters: 1) degree in associative and limbic networks; 2) FC in the sensorimotor, parietal, occipital, and limbic cortex. (Gargouri et al., 2016, Werner et al., 2014) Conversely, two studies described a negative association between motor severity and FC: in left insula, and between postcentral gyrus and occipital fusiform gyrus, between right insula and right supplementary motor area (SMA), and between bilateral caudate nucleus and middle frontal gyrus. (Aracil-Bolaños et al., 2022, Muller et al., 2016) One study found a negative relation between the number of CAG triplets and nodal degree in the posterior cingulate cortex. (Sanchez-Castaneda et al., 2017).

### 3.4 Dystonia

For dystonia, 46 articles were included, (Zhang et al., 2020, Pan et al., 2019, Jochim et al., 2018, Putzel et al., 2018, Mantel et al., 2018, Ni et al., 2017, Huang et al., 2017, Li et al., 2017, Long et al., 2017, Haslinger et al., 2017, Kiyuna et al., 2017, Battistella et al., 2016, Sarasso et al., 2020, Liu et al., 2016, Ren et al., 2015, Bharath et al., 2015, Dresel et al., 2014, Delnooz et al., 2015, Hinkley et al., 2013, Delnooz et al., 2013, Delnooz et al., 2012, Mohammadi et al., 2012, Wei et al., 2021, Jiang et al., 2019, Pan et al., 2021, Feng e al., 2021, de Faria et al., 2020, Norris et al., 2020, Glickman et al., 2020, Mantel et al., 2020, Ma et al., 2021, Corp et al., 2019, Fang et al., 2021, Giannì et al., 2022, Qin et al., 2019, Zito et al., 2022, Kim et al., 2022, Hou et al., 2022, Ekmen et al., 2022, Nieuwhof et al., 2022, Filip et al., 2022, Battistella and Simonyan, 2019, Bianchi et al., 2019, Kita et al., 2018, Fuertinger and Simonyan, 2018, Qin et al., 2018) concerning cervical dystonia (CD; n = 14), writer’s cramp (n = 10), laryngeal dystonia (n = 8), musician's dystonia (n = 5), paroxysmal kinesigenic dyskinesia (n = 6), blepharospasm (n = 6), oromandibular dystonia (n = 2), dyskinetic cerebral palsy (n = 2), spastic cerebral palsy (n = 1), dystonic tremor (n = 1), and generalized dystonia (n = 1). Most studies matched patients and healthy participants for age and gender. The number of patients per study varied from 11 to 83.

#### 3.4.1 Changes in resting-state connectivity

Most studies found differences between groups, except one study performed in 22 paroxysmal kinesigenic dyskinesia patients. (Liu et al., 2016) The most commonly described affected region was the precentral gyrus (left n = 23; right n = 22), followed by the postcentral gyrus (left n = 20; right n = 17), right SMA (n = 18), and left putamen (n = 13), showing both increased and decreased connectivity. Other regions that included but were not limited to the superior frontal and parietal cortex, and thalamus also showed opposite effects (Fig. 1, Fig. 2, Supp. Figs. 2–3). A study in blepharospasm demonstrated that spasm severity co-varied with BOLD connectivity in the sensorimotor cortex and cerebellum, while spasm onset co-varied with BOLD connectivity in the lentiform nucleus and frontal eye field. (Glickman et al., 2020) The ALE analysis (23 and 24 studies for the hyper- and hypoconnectivity contrasts, respectively) showed significant spatial convergence for the hypoconnectivity contrast (Fig. 3), forming two clusters spanning the right precentral and postcentral gyrus, and the bilateral SMA. No significant convergence was found for the hyperconnectivity contrast.

#### 3.4.2 Changes in resting-state networks

Seventeen studies investigated brain networks in patients with dystonia. (Putzel et al., 2018, Mantel et al., 2018, Huang et al., 2017, Haslinger et al., 2017, Battistella et al., 2016, Sarasso et al., 2020, Hinkley et al., 2013, Delnooz et al., 2013, Mohammadi et al., 2012, Glickman et al., 2020, Mantel et al., 2020, Qin et al., 2019, Battistella and Simonyan, 2019, Bianchi et al., 2019, Kita et al., 2018, Fuertinger and Simonyan, 2018, Qin et al., 2018) One study compared CD patients with and without an effective sensory trick with healthy participants, and found that CD-without-trick patients showed increased FC in a SMN, whereas no differences were observed between CD-with-trick patients and healthy participants. (Sarasso et al., 2020) Another study in CD patients found increased FC in a CCN and, in contrast to the previous study, decreased FC in a SMN. (Delnooz et al., 2013).

Using independent component analysis in patients with task-specific focal dystonia revealed increased connectivity in a SMN. (Bianchi et al., 2019) Graph-based network analysis on the same sample (another study) showed abnormal connectivity of motor and somatosensory cortical areas across different TSFD phenotypes, in which each affected body region exhibited distinct aberrant connectivity, namely, hand dystonia involving motor execution, and laryngeal dystonia involving sensorimotor processing. (Fuertinger and Simonyan, 2018) Two studies done in TSFD (writer’s cramp) found decreased FC in a SMN, while the latter study additionally found increased FC between the putamen and the cortical regions of the DMN. (Hinkley et al., 2013, Mohammadi et al., 2012) Another study described that, beyond FC increases within cerebellum-cortical and basal ganglia-cortical networks, reduced FC was found between these networks in writer’s cramp. (Mantel et al., 2018).

In SD, one study reported both increased and decreased FC in a SMN (sensorimotor and inferior parietal cortices), (Putzel et al., 2018) while two other studies found decreased FC in a SMN. (Battistella et al., 2016, Battistella and Simonyan, 2019) The latter additionally showed increased FC in a CCN. (Battistella et al., 2016, Battistella and Simonyan, 2019) Increased FC in a SMN has also been reported together with increased FC in an auditory network. (Mantel et al., 2020) Regarding musician’s dystonia, increased FC in a basal ganglia network (putamen) has been described. (Kita et al., 2018) Furthermore, in embouchure dystonia, abnormal connectivity of sensorimotor representations of affected and unaffected body parts was found, together with an auditory network change, as well as altered connectivity to a cerebellar network. (Haslinger et al., 2017).

In dyskinetic cerebral palsy, decreased connectivity was observed in a cerebellar, SMN, CCN, and SN. (Qin et al., 2018) Furthermore, CCN and SN showed decreased functional asymmetry in children with this disorder. (Qin et al., 2019) In blepharospasm, decreased FC within a SMN and FPN, and increased FC with a SN was found. (Huang et al., 2017) In paroxysmal kinesigenic dyskinesia patients with a *PRRT2* gene mutation, decreased FC within a DMN was found. (Ekmen et al., 2022) Overall, it can be noted that there are inconsistent results with regards to resting-state networks in patients with dystonia.

#### 3.4.3 Correlation with clinical variables

Of the 26 studies investigating the relationship between clinical measures and resting-state brain connectivity, 10 found no significant correlation. Eight studies describe positive relationships between motor severity and brain function: 1) FC between the left and right calcarine cortex and in the SMA; (Qin et al., 2019) 2) fALFF and ReHo in the caudate nucleus; (Ni et al., 2017) 3) FC between both caudate nuclei; (Ni et al., 2017) 4) FC between thalamus and caudate nucleus; (Kiyuna et al., 2017) 5) FC between precentral gyrus and insula, dorsolateral prefrontal cortex, postcentral gyrus, and thalamus; (Dresel et al., 2014) 6) ReHo in cerebellum; (Wei et al., 2021) 7) FC between prefrontal cortex and postcentral gyrus, and between postcentral gyrus and caudate nucleus; (Feng, 2021) 8) connectivity in the cerebellum; (Glickman et al., 2020) 9) FC in supramarginal gyrus and postcentral gyrus. (Ma et al., 2021) Conversely, three studies describe negative relationships between motor severity and brain function: 1) FC between both SMA; (Jiang et al., 2019) 2) FC between cerebellum and visual cortex; (Jochim et al., 2018) 3) FC in the superior parietal cortex. (Delnooz et al., 2012).

Three studies reported positive correlations between disease duration and brain function: 1) FC between thalamus and right precentral gyrus; 2) FC between cerebellum and sensorimotor and premotor cortex; 3) FC between bilateral thalamus and bilateral precentral gyrus. (Long et al., 2017, Dresel et al., 2014, Kim et al., 2022) Conversely, three studies reported negative correlations between disease duration and brain functioning: 1) FC in right superior frontal gyrus; 2) FC between left and right putamen, and between left and right insula; 3) FC in left superior parietal cortex. (Huang et al., 2017, Ren et al., 2015, Delnooz et al., 2012).

A positive correlation between age of onset and nodal efficiency of the pallidum has been reported, as well as a negative correlation between age of onset and FC in the left inferior parietal cortex. (Zhang et al., 2020, Putzel et al., 2018).

### 3.5 Functional movement disorders

Eleven articles on FMD patients were included in this review. (Canu et al., 2020, Marapin et al., 2020, Mueller et al., 2022, Marapin et al., 2021, Wegrzyk et al., 2018, Diez et al., 2019, Morris et al., 2017, Baek et al., 2017, Maurer et al., 2016, Spagnolo et al., 2020, Piramide et al., 2022) Patients were diagnosed with several FMD phenotypes, including functional tremor, functional jerks, functional dystonia, and functional weakness. All studies compared patient groups with age- and gender-matched healthy participants. The number of patients per study varied from 17 to 40.

#### 3.5.1 Changes in resting-state connectivity

All studies found significant differences in resting-state connectivity between patients and healthy participants. The most commonly affected brain regions included the right middle frontal gyrus (n = 3), inferior parietal cortex, (n = 3), left cerebellum lobule VI (n = 3), right insula (n = 3), left precuneus (n = 3), and the right SMA (n = 3). While the first two regions showed mixed results, the cerebellum and right insula consistently showed increased and decreased connectivity, respectively. Other reported regions include but are not limited to the amygdala, insula, SMA, and parietal cortex, showing opposite effects (Fig. 1, Fig. 2, Supp. Figs. 2–3). A study investigating the contribution of genetic polymorphisms found that patients with the tryptophan hydroxylase 2–703 G/T polymorphism showed decreased FC between the amygdala and middle frontal gyrus, compared with GG homozygotes and healthy participants. (Spagnolo et al., 2020) The ALE analysis (four and seven studies for the hyper- and hypoconnectivity contrasts, respectively) showed significant spatial convergence for the hypoconnectivity contrast (Fig. 3), forming a cluster spanning the right supramarginal and angular gyrus, whereas no significant convergence was found for the hyperconnectivity contrast.

#### 3.5.2 Changes in resting-state networks

One study found aberrant connectivity in a DMN and CCN in patients with functional tremor and functional myoclonus. (Marapin et al., 2021

#### 3.5.3 Correlation with clinical variables

Of the seven studies investigating the correlation between clinical measures and resting-state brain connectivity, two found no significant correlation. (Marapin et al., 2020, Marapin et al., 2021) Two studies reported a positive relationship between motor severity and: 1) FC between left and right insula and temporoparietal junction (TPJ); 2) connectivity in the precuneus and bilateral temporoparietal junction. (Mueller et al., 2022, Diez et al., 2019) On the other hand, a negative correlation was reported for motor severity and FC between the right amygdala and right middle frontal gyrus. (Spagnolo et al., 2020) One study described a positive relationship between disease duration and FC between the ventromedial prefrontal cortex and thalamus. (Canu et al., 2020) Finally, a study investigating the effect of childhood emotional abuse found a positive correlation with the FC between the right TPJ and left insula. (Maurer et al., 2016).

### 3.6 Myoclonus

For myoclonus, 11 articles were included. (Wang et al., 2020, Kim et al., 2019, Yang et al., 2022, Wang et al., 2019, Jia et al., 2018, Zhong et al., 2018, Jiang et al., 2018, Long et al., 2016, Jiang et al., 2016, Ur Özçelik et al., 2021, Zhang et al., 2020) Patients were diagnosed with juvenile myoclonic epilepsy (JME; n = 9) and familial cortical myoclonic tremor with epilepsy (n = 2). Most patients were compared with age- and gender-matched healthy participants. The number of patients per study varied from 11 to 75.

#### 3.6.1 Changes in resting-state connectivity

All studies found significant differences between groups. The most affected brain areas were the thalamus (left n = 6; right n = 5), left anterior cingulate cortex (n = 4), right precuneus (n = 4), left cerebellum crus 1 and lobule VIII (n = 3), and right middle temporal gyrus (n = 3). Notably, all studies implicating the left thalamus, anterior cingulate cortex and middle temporal gyrus consistently showed increased connectivity. Other described regions include but are not limited to the middle frontal and parietal cortex, showing opposite effects (Fig. 1, Fig. 2, Supp. Figs. 2–3). The ALE analysis (ten and seven studies for the hyper- and hypoconnectivity contrasts, respectively) showed significant spatial convergence for both contrasts (Fig. 3). For the hyperconnectivity contrast, significant clusters were found in the bilateral putamen, whereas for the hypoconnectivity contrast clusters were found in the left caudate and right precuneus and cuneus.

#### 3.6.2 Changes in resting-state networks

One study found changes in regions of the DMN in 34 patients with JME. (Zhang et al., 2020).

#### 3.6.3 Correlation with clinical variables

Of the seven studies investigating the relationship between clinical measures and resting-state brain connectivity, three studies found no significant correlation. (Kim et al., 2019, Jia et al., 2018, Long et al., 2016) Two studies described positive relationships between the age of onset and brain function: 1) FC between the right caudate nucleus and right cerebellum; 2) ReHo in the left paracentral lobule. (Zhong et al., 2018, Jiang et al., 2016) One study found a negative relation between age of onset and FC between the left putamen and right superior occipital gyrus. (Zhong et al., 2018) Two studies described positive correlations between disease duration and brain function: 1) ALFF in the left cerebellum; 2) FC between thalamus and bilateral lingual gyrus and right pallidum. (Wang et al., 2020, Jiang et al., 2018) Conversely, one study found a negative correlation between disease duration and FC between both superior frontal gyri and between right hippocampus and right inferior frontal gyrus. (Zhong et al., 2018).

### 3.7 Tics

Nineteen articles on patients with tics were included, (Bhikram et al., 2020, Openneer et al., 2020, Tinaz et al., 2014, Neuner et al., 2014, Shprecher et al., 2014, Cui et al., 2014, Church et al., 2009, Lou et al., 2020, Tikoo et al., 2020, Worbe et al., 2012, Zito et al., 2021, Ramkiran et al., 2019, Fan et al., 2018, Liu et al., 2017, Wen et al., 2018, Liao et al., 2017, Greene et al., 2016, Tinaz et al., 2015, Ganos et al., 2014) all concerning Tourette syndrome (TS). Most patients were compared with age- and gender-matched healthy participants. The number of patients per study varied from 10 to 79.

#### 3.7.1 Changes in resting-state connectivity

Except for two studies, (Tinaz et al., 2014, Greene et al., 2016) changes in brain function were most commonly reported in the caudate nucleus (right n = 4; left n = 3), putamen (right n = 4, left n = 3), cerebellar crus 1 (n = 3), insula (n = 3), bilateral anterior cingulate cortex (n = 3), and thalamus (n = 2). The cerebellum, insula, and putamen were consistently involved. Other described regions include but are not limited to the superior frontal, parietal, and temporal cortex, showing mixed results (Fig. 1, Fig. 2, Supp. Figs. 2–3). Investigating the neural correlates underlying voluntary tic inhibition revealed an association with increased connectivity in the left inferior frontal gyrus, which differs from premonitory urges. (Ganos et al., 2014) The origin and temporal pattern of tic generation has been described to follow cortico-striato-thalamo-cortical circuitry, in which cortical areas precede subcortical activation. (Neuner, 2014) The ALE analysis (7 studies each for the hyper- and hypoconnectivity contrasts) did not show any statistically significant clusters in both contrasts.

#### 3.7.2 Changes in resting-state networks

Decreased local efficiency and clustering coefficient values have been found in the DMN. (Openneer et al., 2020) Additionally, others found a reduction in average path length within a DMN and SN. (Ramkiran et al., 2019) In contrast, increased FC in a DMN has also been reported, together with decreased FC within a CCN, as well as increased FC between DMN and the CCN. (Fan et al., 2018) Others found increased FC within a CCN. (Lou et al., 2020) Showing similar results, increased FC in a basal ganglia, cerebellar, SMN, and DMN has also been found, together with decreased FC in a CCN and SN. (Tikoo et al., 2020) Finally, one study found a decreased characteristic path length and increased network strength in sensorimotor, associative, and limbic networks. (Worbe et al., 2012).

#### 3.7.3 Correlation with clinical variables

Of the 15 studies investigating the correlation between clinical measures and resting-state brain connectivity, two found no significant correlation. (Tinaz et al., 2014, Zito et al., 2021) Five studies describe positive relationships between motor severity and brain function: 1) FC between the caudate nucleus and superior occipital gyrus, and between putamen and precentral and postcentral gyrus (Bhikram et al., 2020); 2) centrality of the right parahippocampal gyrus, left middle temporal gyrus, and left Heschl’s gyrus (Wen et al., 2018); 3) network strength in the SMA (Neuner, 2014); 4) fALFF in the right thalamus (Cui et al., 2014); 5) network strength in sensorimotor, associative, and limbic networks. (Worbe et al., 2012) On the other hand, five studies describe negative correlations between motor severity and brain function: 1) FC within a DMN (Fan et al., 2018); 2) FC between both anterior cingulate cortices (Liao et al., 2017); 3) ReHo in the right operculum (Lou et al., 2020); 4) FC in right cerebellum (Tikoo et al., 2020); 5) characteristic path length in sensorimotor, associative, and limbic networks. (Worbe et al., 2012).

One study found a correlation between urge severity and FC between the right insula and bilateral SMA. (Tinaz et al., 2015) Furthermore, another study found a positive correlation between the ability of patient to inhibit tics and ReHo in the left inferior frontal gyrus. (Ganos et al., 2014) With regards to disease duration, two studies found a positive relationship with brain functioning: 1) ReHo of the right cerebellum; 2) centrality of left Heschl’s gyrus and right superior frontal gyrus. (Liu et al., 2017, Wen et al., 2018).

### 3.8 Tremor

The 51 included articles on tremor concerned tremor-dominant Parkinson’s disease (TDPD; n = 28), essential tremor (ET; n = 21), orthostatic tremor (n = 2), and primary writing tremor (n = 1). (Bagarinao et al., 2022, Basaia et al., 2022, Benito-Leon et al., 2015, Benito-Leon et al., 2016, Benito-Leon et al., 2019, Chen et al., 2015, Chen et al., 2019, Dirkx et al., 2017, Dirkx et al., 2019, Duan et al., 2021, Fang et al., 2013, Fang et al, 2015, Fang et al., 2016, Gallea et al., 2015, Gallea et al., 2016, Gu et al., 2017, Helmich et al., 2011, Hou et al., 2018, Hu et al., 2015, Hu et al., 2017, Hu et al., 2019, Jiang et al., 2016, Karunanayaka et al., 2016, Kato et al., 2022, Lenka et al., 2017, Lenka et al., 2017, Li et al., 2018, Li et al., 2020, Li et al., 2021, Li et al., 2021, Ma et al., 2015, Ma et al., 2017, Mueller et al., 2017, Nicoletti et al., 2020, Nigro et al., 2019, Popa et al., 2013, Quarantelli et al., 2022, Tikoo et al., 2020, Tuleasca et al., 2018, Tuleasca et al., 2020, Vervoort et al., 2016, Wang et al., 2016, Wang et al., 2018, Wang et al., 2021, Wen et al., 2016, Yin et al., 2016, Zhang et al., 2015, Zhang et al., 2015, Zhang et al., 2015, Zhang et al., 2016, Zhu et al., 2021) The number of patients per study ranged from 10 to 99.

#### 3.8.1 Changes in resting-state connectivity

Most studies found differences between patients and healthy participants, except one study on TDPD. (Karunanayaka et al., 2016) The most commonly affected brain regions included the bilateral precentral gyrus (left n = 19; right n = 17), right cerebellar crus 1 (n = 17), bilateral SMA (n = 17), bilateral cerebellum lobules IV-V (n = 16), right middle frontal gyrus (n = 16), bilateral superior frontal gyrus (n = 12), and bilateral thalamus (left n = 11; right n = 10). Both increased and decreased connectivity were observed. Other reported regions included but were not limited to the bilateral postcentral gyrus, insula, and several cerebellar regions, also showing mixed results (Fig. 1, Fig. 2, Supp. Figs. 2–3). The ALE analysis (28 and 29 studies for the hyper- and hypoconnectivity contrasts, respectively) showed significant spatial convergence for the hypoconnectivity contrast (Fig. 3), forming a cluster in the right cerebellum (lobules IV-V), whereas no significant convergence was found for the hyperconnectivity contrast.

A study comparing patients with dopamine-responsive and dopamine-resistant tremor found that the latter had increased tremor-related connectivity in non-dopaminergic areas (cerebellum) and reduced cortical somatosensory influences on the thalamus, which in turn was less sensitive to dopamine. (Dirkx et al., 2019) Others tested the effect of dopamine on connectivity in TDPD, and concluded that dopamine controls PD tremor by acting on the cerebellar thalamus and pallidum. (Dirkx et al., 2017) A study done in patients with PD found that tremor scores mainly correlated with cerebellar connector hubs, whereas postural instability/gait difficulty scores primarily correlated with sensorimotor connector hubs. (Bagarinao et al., 2022) Finally, a study assessed if the severity of nigrostriatal innervation loss affects the FC of the sensorimotor cortico-striatothalamic-cortical loop in ET and PD. (Quarantelli et al., 2022) This was not the case in ET, whereas in PD patients the putamen dopamine intake showed an inverse correlation with the FC of thalamus and cerebellum with sensorimotor cortices.

#### 3.8.2 Changes in resting-state networks

Investigating the dynamic FC of resting-state networks in ET revealed impaired functioning of a “cerebello-visuo-motor” network, a “thalamovisuomotor network” and a “basal ganglia and extrastriate” network. (Tuleasca et al., 2020) Another study in ET patients showed increased connectivity in a DMN and CCN, and decreased connectivity in cerebellar and visual networks. (Benito-Leon et al., 2015) In line with the previous finding, decreased connectivity in a cerebellar network, as well as increased connectivity in a SMN and SN has been reported in ET patients. (Fang et al., 2015) Additionally, increased FC was observed between an anterior and posterior DMN, and decreased functional network connectivity between a cerebellum network and a SMN and posterior DMN. Furthermore, a study described decreased FC within a SMN, visual network, and visuo-spatial network. (Kato et al., 2022) In orthostatic tremor, findings similar to a study done in ET patients were reported, namely increased connectivity in a DMN and CCN, and decreased connectivity in a cerebellar and SMN. (Benito-Leon et al., 2016).

A graph-based network analysis in patients with TDPD found decreased local efficiency and increased global efficiency in the functional brain network. (Zhang et al., 2015) Another study in TDPD patients found increased local and global efficiency in a tremor-related network, consisting of cortical (sensorimotor cortex, prefrontal and occipital areas), subcortical (thalamus and basal), and regions in the cerebellum and brainstem. (Zhang et al., 2015) Finally, a dynamic FC study found increased FC between a SMN and CCN in patients with TDPD. (Zhu et al., 2021).

#### 3.8.3 Correlation with clinical variables

Due to the vastness of results, we only report correlations between brain function and motor severity. Of the 31 studies investigating the correlation between clinical measures and resting-state brain connectivity, four studies did not find significant associations. (Benito-Leon et al., 2019, Chen et al., 2019, Hu et al., 2019, Li et al., 2021) In ET, six studies describe positive relationships between motor severity and brain function: 1) ALFF in left cerebellum lobule VIII and right cerebellum lobule VI; (Wang et al., 2018) 2) connectivity in the bilateral putamen, and connectivity between the pre-SMA and putamen, as well as the connectivity between the bilateral ventral intermediate nucleus and right putamen; (Mueller et al., 2017) 3) FC between bilateral thalami and right cerebellum lobule IX; (Lenka et al., 2017) 4) FC in left fusiform gyrus; (Benito-Leon et al., 2015) 5) FC in bilateral sensorimotor cortex; (Fang et al., 2015) 6) FC between precentral gyrus and bilateral posterior parietal areas, and FC between thalamus and bilateral cerebellum. (Nicoletti et al., 2020).

Conversely, six studies describe negative relationships between motor severity and brain function in ET: 1) FC between dentate nucleus and several brain regions, including the left sensorimotor cortex, left SMA, and bilateral superior parietal lobes; (Tikoo et al., 2020) 2) FC between left postcentral gyrus and right cerebellum crus 1; (Lenka et al., 2017) 3) FC in bilateral cerebellum lobules IV-V and left cerebellum lobule VI; (Fang et al., 2015) 4) ALFF in bilateral SMA and precentral gyrus; (Gallea et al., 2015) 5) FC between the precentral gyrus and bilateral premotor areas, bilateral SMA, right thalamus and bilateral cerebellum crus 1, lobule VI, and left cerebellum crus 2; (Nicoletti et al., 2020) FC in the left precentral gyrus. (Kato et al., 2022).

In TDPD, two studies describe positive relationships between motor severity and brain function: 1) ReHo in the left inferior parietal lobule, left cerebellum lobule IV-V, and cerebellum vermis 3; (Wen et al., 2016) FC between left ventral intermediate nucleus and left precentral gyrus, bilateral cerebellum, and left postcentral gyrus. (Zhang et al., 2016) On the other hand, four studies describe negative relationships between motor severity and brain functioning: 1) ReHo in bilateral putamen, right cerebellum crus II and lobule IV-V, and right SMA; (Li et al., 2020) 2) ReHo in the right caudate nucleus and right precuneus; (Wen et al., 2016) 3) network local efficiency; (Zhang et al., 2015) 4) FC between the left and right cerebellum lobule VIII. (Hu et al., 2015).

In primary writing tremor, a negative relationship was reported between motor severity and clustering coefficient of the bilateral medial cerebellum and left posterior parietal cortex. (Lenka et al., 2017).

The commonalities and differences in implicated brain regions across the HMD phenotypes is shown in Fig. 4.

## 4 Discussion

Brain regions with impaired connectivity, as well as the direction of these alterations, were described and visually demonstrated in this systematic review (Fig. 1, Fig. 2, Supp. Figs. 2–3). Altered connectivity profiles were found in distinct, as well as overlapping brain regions across HMD. When assessing whether or not these connectivity profiles (i.e., hyperconnectivity or hypoconnectivity) were consistently reported across studies, for many phenotypes significant convergence in brain regions could not be identified. Significant spatial convergence was only found for dystonia, FMD, myoclonus, and tremor (Fig. 3). The lack of significant findings for the other phenotypes should not be fully surprising, given the fact that many phenotypes (i.e., ataxia, chorea, FMD, myoclonus, and tics) included <15 studies per phenotype. For these phenotypes, the analyses were not adequately powered, as the recommendation is to have at least 17–20 studies to achieve a 80 % power. (Cortese et al., 2021) Furthermore, in this study we included studies that investigated a diverse array of rs-fMRI measures, which could explain the lack of convergence for some phenotypes, similar to Cortese et al. (Cortese et al., 2021). Finally, the lack of spatial convergence may stem from the fact that precise locations of aberrant connectivity within brain circuits may well vary across individuals and phenotypes, making it difficult to discover spatial convergence across studies.

A presented heat map summarized commonly and differentially implicated brain regions across HMD phenotypes (Fig. 4), which shows that chorea and tics are commonly characterized by impaired subcortical connectivity, whereas altered connectivity in FMD is mainly found in the cognitive control network. Ataxia is primarily characterized by aberrant connectivity in the cerebellar network, while tremor, myoclonus, and FMD also show aberrations in this network. For dystonia, altered connectivity was mainly described in the sensorimotor network, which to a lesser extent was also the case for tremor and chorea. Here, it is important to note that results in opposite directions (i.e., both decreased and increased resting-state BOLD signal connectivity) were found for all HMD. These discrepancies in connectivity profiles could particularly be the result of different methods being used (e.g., FC vs ALFF analysis), as well as clinical differences (i.e., early vs advanced disease status) between investigated patient cohorts for each HMD phenotype. Finally, correlations with several clinical features were described.

There are eight methodological issues which need to be taken into account when interpreting the results presented in this review. First, the direction of the results (Supp. Figs. 2–3) should be interpreted with caution. Namely, we determined the direction of a finding *per study* based on whether a brain region was found to be altered, along with its effect size. In some studies, there were results in opposite directions within the same study and AAL brain region. For these studies, the affected brain regions were not assigned an effect size, as we did not want to consider ambivalent results. This meant that some data was discarded. Importantly, a region can exhibit both increased (with region X) and decreased connectivity (with region Y). Discarding regions for the above listed reason is consistent with the aim of this study, i.e., to provide a general overview of the regions with an altered network contribution in rs-fMRI studies on HMD, and not to precisely characterize and quantify the interactions between such brain regions.

Second, as many studies investigated *a priori* defined brain regions in accordance with their hypotheses, many regions were not considered and could have thus influenced our findings. Third, labelling of the involved regions differed between articles. To provide a visual overview (Fig. 1, Fig. 2, Supp. Figs. 2–3), all regions were transformed into the AAL atlas. However, the absence of exact coordinates in some studies makes this transformation prone to subjectivity. In addition, the definition of networks also has a subjective component. This subjectivity could potentially contribute to the inconsistent findings with regards to altered network connectivity. Fourth, we included studies using different analysis methods (e.g., ReHo, ALFF, FC, graph analysis), as long as they investigated resting-state signal fluctuations and synchronization, to provide the most comprehensive overview of resting-state abnormalities in HMD. However, the relevance of some of these measures remain disputed and need to be interpreted carefully. Furthermore, while these methods are postulated to reflect neural activity, their different neurophysiological bases and underlying associations may affect the results of this review.

Fifth, in the absence of longitudinal data, in general it is challenging to identify whether the here described alterations found in HMD using rs-fMRI are primary, secondary, or compensatory. Sixth, although the spatial resolution of rs-fMRI is good (2–3 mm), small regions that are of interest in HMD such as brainstem and thalamic nuclei may have remained undetected. Seventh, many studies included a relatively small number of patients, this might have had an influence on the results due to a lack of power. However, small sample sizes are inevitable since most HMD are relatively rare. Therefore, to give a generalizable overview, we used the standardized mean difference as a measure of effect size to report our results. Finally, for the ALE analysis, we only considered studies that reported significant result with their coordinates. However, many studies did not report the coordinates of their findings in their work. Therefore, a limitation is that not all results could be considered for this meta-analysis.

Resting-state fMRI studies on **ataxia** included several disorders, and primarily found alterations in the cerebellum. The cerebellum is a key region in the pathophysiology of ataxia, and its putative functions include motor learning and adaptation, coordination, and modulation of sensorimotor gain. (Marsden and Harris, 2011) Unsurprisingly, cerebellar damage therefore gives rise to the classic motor signs and symptoms often seen in patients with ataxia. Interestingly, other regions also seem to be involved in ataxia disorders, including regions in the frontal, parietal, and temporal cortex. Furthermore, abnormalities in the cerebellar network, as well as the default mode network, frontoparietal network, and visual network were described in these disorders. These results likely reflect the spectrum of both motor and non-motor deficits that is commonly seen in spinocerebellar ataxias and other ataxia disorders. Due to the small sample size of included studies, however, these results should be interpreted with caution.

All rs-fMRI studies in **chorea** investigated patients with Huntington’s disease. The caudate nucleus (striatum) and SMA are among the most frequently implicated brain regions. This is consistent with the hypothesized pathology of chorea which points to a dysfunction of a complex neuronal network consisting of the basal ganglia and different motor cortical areas. (Cardoso et al., 2006) Interestingly, the caudate nucleus showed both increased and decreased connectivity. These opposite findings could be due to different methods used, a dynamic pattern of connectivity, or due to the nature of this review as described above. Due to the clinical heterogeneity found in HD, it is unsurprising that impaired connectivity was also found in the superior frontal cortex, parietal, and inferior temporal cortex. Furthermore, impaired connectivity was found in the default mode, sensorimotor, associative, and limbic networks. The involvement of these networks supports the hypothesis that the dysfunction of different networks might contribute to the clinical heterogeneity of HD. (Gargouri et al., 2016) Again, due to the small sample size of included studies, these results should be carefully interpreted.

The rs-fMRI studies on **dystonia** included a variety of disorders, mainly cervical dystonia, writer’s cramp, and laryngeal dystonia. The most commonly described affected regions included the sensorimotor cortex, SMA, putamen, parietal cortex, and thalamus. The cerebellum also seems to be implicated (Fig. 1, Fig. 2, Supp. Figs. 2–3). This is in accordance with the main pathophysiological model which points to dysfunction of the basal ganglia and sensorimotor circuits, including, more recently, the cerebello-thalamo-cortical pathways. (Vidailhet et al., 2012) While opposite results were found in these regions, they nevertheless mainly exhibited negative connectivity, with the exception of the putamen (Fig. 1, Fig. 2, Supp. Figs. 2–3). The ALE analysis yielded two clusters of convergence, consisting of the right precentral and postcentral gyrus and the bilateral SMA. This is in line with the fact that these regions were also the most commonly reported regions across studies. However, convergence was not found for several other key regions implicated in dystonia, notably the basal ganglia. This may reflect a number of factors, such as the fact that this analysis may still be insufficiently powered and therefore not detect all significant effects. While the number of studies was higher than the recommended minimum (of n = 17), this is not a gold standard threshold. Therefore, a larger number of studies is probably needed to detect all significant effects, possibly with small to medium effect sizes. (Cortese et al., 2021) Another important factor in this regard is that studies investigating different dystonia subtypes (e.g., cervical dystonia, writer’s cramp, laryngeal dystonia) were all synthesized and considered as one phenotype (dystonia) for the ALE analysis, making this a heterogeneous group. This heterogeneity could therefore potentially account for the fact that several key brain regions (known from the literature) were not found in this analysis. Nevertheless, the aim of this study was to identify affected brain regions for the main HMD phenotypic categories, and not the different subtypes, to better understand the overall brain connectivity profiles for each HMD.

Other possible factors underpinning this could be due to heterogeneity of the studies included, i.e., heterogeneous participant characteristics (age, sex), MRI parameters, analysis procedures (statistical thresholding, head-motion artefact mitigation strategies). Finally, there is also the possibility that the hypothesized affected brain regions in dystonia which did not show significant spatial convergence (e.g., putamen) in rs-fMRI studies are not characterized by abnormal intrinsic brain activity during rest. In this regard, an ALE analysis on task-based fMRI studies could be useful, to determine whether these brain regions do converge during motor tasks.

Several networks were found to be affected, mainly the sensorimotor, cerebellar, basal ganglia, cognitive control, and default mode network, with different changes for each network. Altered connectivity between sensorimotor, cerebellar, and basal ganglia networks underscores the important role of these structures in dystonia. With regards to the role of the default mode network, the authors of that study hypothesized that increased connectivity of the putamen with the default mode network may reflect dysfunction of cortico-subcortical circuits in writer’s cramp. (Mohammadi, 2012) Interestingly, in both adductor-type spasmodic dysphonia and embouchure dystonia, auditory network abnormalities were found, which could reflect a possible subtype associated component. I.e., it can possibly be interpreted as an expression of maladaptive rewiring that, beyond the sensorimotor circuits, affects multiple modalities including auditory networks. A close link between auditory and sensorimotor input is pivotal for musical performance (embouchure dystonia), and the auditory network has been suggested to be part of the phonation network, which is impaired in adductor-type spasmodic dysphonia. (Haslinger et al., 2017, Mantel et al., 2020).

Resting-state fMRI studies on **FMD** included patients with several FMD phenotypes, including functional tremor, functional jerks, functional dystonia, and functional weakness. Frequently implicated regions include the frontal and parietal cortex, insula, and cerebellum. Interestingly, the cerebellum consistently showed increased connectivity, which has also been shown in other neuroimaging studies in FMD. (Sasikumar and Strafella, 2021) Furthermore, a study showed impaired functioning of areas of the dorsal attention network and default mode network. The dorsal attention network has a close relationship with sensorimotor regions and plays a key role in visuospatial perceptual attention, e.g., shifting of attention to salient objects in the external environment. (Dixon et al., 2018) The default mode network, on the other hand, is involved in mentalizing, self-referential processing, and emotional processing. The reviewed fMRI studies thus indicate impairment of these functions in FMD. Crucially, Spagnolo et al. are the first study investigating the contribution of stress-related genetic polymorphisms to the clinical and circuit-level phenotype of FMD. They describe that the TPH2 genotype may modulate FMD both directly and interactively with childhood trauma, and suggest that their findings support a potential molecular mechanism modulating FMD.

Patients with FMD have several abnormalities in their neurobiology, including heightened connectivity between motor and limbic networks. (Baizabal-Carvallo et al., 2019) Furthermore, there is altered top-down regulation of motor functions and increased activation of areas implicated in self-awareness, self-monitoring, and active motor inhibition, such as the cingulate and insular cortex. Furthermore, impaired sense of agency, the feeling that one is in control of one's own actions, is a characteristic feature of FMD. (Edwards et al., 2012) The temporoparietal junction area has been highlighted as the neural correlate of sense of agency. (Haggard, 2017) The rs-fMRI findings are thus in line with the postulated mechanisms underlying FMD. Importantly, the ALE analysis showed a significant cluster in the right TPJ (angular gyrus), further supporting these findings. Furthermore, motor severity correlated with connectivity in the prefrontal and insular cortex, TPJ, amygdala, and thalamus. Due to the small sample size of included studies, caution is necessary in the interpretation.

Resting-state fMRI studies on **myoclonus** mainly involved patients with JME, except for two studies in familial cortical myoclonic tremor with epilepsy. Notably, studies on isolated myoclonus are scant, as most studies report on patients who also suffer from epilepsy, and may have influenced the results. Alterations in the thalamus, cerebellum, cingulate, primary motor, and temporal cortex were most frequently mentioned. Notably, all studies implicating the thalamus, cingulate cortex, and temporal cortex consistently showed increased connectivity. These findings fit the notion that myoclonus is the result of enhanced excitability of the sensorimotor cortex generated by processes involving cerebellar pathology and alterations of the cerebello-thalamo-cortical pathways. (Ganos et al., 2014, Tijssen et al., 2000) Moreover, the epileptiform discharges seen in JME has been linked to temporal cortex connectivity. (Holmes et al., 2010) Interestingly, while the ALE analysis found significant effects for the precuneus and basal ganglia, this was not found for the thalamus, which was unexpected given that the thalamus was the most commonly implicated brain region. This could also be related to the aforementioned explanations in the paragraph discussing dystonia.

One network study found changes in regions of the default mode network in patients with JME, which has been suggested to reflect an important role of emotional dysregulation in the development of JME, given the putative functional roles of the affected default mode network structures. (Zhang et al., 2020) Previous studies have also implicated these structures in JME patients, perhaps most interestingly a task-based fMRI study which found impaired default mode network connectivity. (Vollmar, 2011) In that study, the authors reason that their observations in JME could possibly be interpreted as an imbalance between the task relevant cognitive network and the opposing default mode network. I.e., an ‘overload’ of the task relevant cognitive network during a cognitively demanding task, together with impaired deactivation of the default mode network, could lead to hyperexcitability across brain regions, including the motor cortex, and cause myoclonic jerks. Due to the small sample size of included studies, caution is necessary in interpretation here.

All studies concerning **tics** were conducted in patients with Tourette’s syndrome. The most frequently reported regions included the striatum, cerebellum, insula, cingulate cortex, and thalamus. Notably, the cerebellum consistently showed decreased connectivity, whereas the insula and putamen consistently showed increased connectivity. This is in line with the evidence that points to a disorder of the basal ganglia-thalamo-cortical and limbic circuits in TS, (Berardelli et al., 2003, Caligiore et al., 2017, Worbe et al., 2015) fitting the model that tics originate from abnormal basal ganglia neuronal discharges that lead to thalamocortical overactivity, causing the involuntary hyperkinetic movements. Interestingly, a study demonstrated that the origin and temporal pattern of tic generation follows cortico-striato-thalamo-cortical circuitry, in which cortical areas precede subcortical activation. (Neuner, 2014) It has been postulated that the failure of top-down cortical regulation over motor pathways could be a possible mechanism of tic genesis. (Worbe et al., 2015).

Several resting-state networks were described to be affected in TS, namely a basal ganglia network, default mode network, cognitive control, salience network, sensorimotor network, associative, and limbic networks, showing mixed results. Specifically, the involvement of the default mode network and salience network have been linked to the premonitory urge feeling which typically precedes the onset of tics in patients with TS. (Ramkiran et al., 2019) Importantly, the discrepancy in network findings could result from the fact that network impairments are dependent on clinical levels of disease expression in TS, which could have differed across patient cohorts in this review.

The rs-fMRI studies on **tremor** mainly involved patients with ET and TDPD, except for one study investigating orthostatic tremor and one study investigating primary writing tremor. The most commonly described affected brain regions included the cerebellum, sensorimotor cortex, SMA, prefrontal cortex, thalamus, and insula. These findings are in line with pathophysiological models implicating the cerebello-thalamo-cortical circuitry in both ET and TDPD. (Helmich et al., 2013, van der Stouwe et al., 2020) It has been postulated that, in ET, the cerebellum likely acts as an oscillator, potentially due to the loss of inhibitory mechanisms. In contrast, in TDPD, the cerebellum may be a modulator which contributes to tremor oscillations by influencing the thalamocortical circuit. (van den Berg and Helmich, 2021) The role of the cerebellum in tremor is further underscored by the ALE findings of this study, namely implicating the cerebellum lobule IV-V, a region to which sensorimotor regions project and which has been shown to be active during a finger-tapping task. (Stoodley et al., 2012) Notably, in a study investigating FC of the dentate nucleus, connectivity with the cerebellum and caudate was found to be increased and decreased, respectively, in TDPD patients. (Ma et al., 2015) Another study in TDPD with dopamine-resistant tremor found increased tremor-related connectivity in the cerebellum. Furthermore, dopamine increases thalamic inhibition to a greater extent in dopamine-responsive tremor compared to dopamine-resistant tremor. (Dirkx et al., 2019) Another study showed that dopamine controls Parkinson’s tremor by acting on the cerebellar thalamus and pallidum, but not in the cerebellum or primary motor cortex. (Dirkx et al., 2017).

In ET, a study investigating FC of the dentate nucleus found decreased FC with cortical, subcortical, and cerebellar areas. (Tikoo et al., 2020) Furthermore, they found that tremor severity and disease duration correlated negatively with FC between the dentate nucleus and SMA, sensorimotor and prefrontal cortex. In addition, dentate nucleus FC with the cerebellar cortex and thalamus was positively and negatively correlated with tremor amplitude, respectively. Taken together, this study suggests that a functional disconnection of the dentate nucleus is involved in the pathophysiology of ET, supporting the cerebellar decoupling hypothesis. (van der Stouwe et al., 2020).

Several networks were implicated in studies on tremor, such as a default mode, cognitive control, sensorimotor, salience, cerebellar, and visual network. Changes in the sensorimotor and cerebellar network support the notion that the CTC circuit is indeed impaired in ET. The default mode, cognitive control, and salience network are believed to be involved in cognitive functioning, and dysfunction in these network could underly the nonmotor manifestations (e.g., cognitive impairment) frequently associated with ET. A study in TDPD found impaired FC between the sensorimotor and cognitive control network. The SMA is a key region of the sensorimotor network and is related to the planning and execution of voluntary movements. (Lee et al., 1999) Moreover, the cognitive control network contributes to executive control, the ability to voluntarily guide action based on goals. Therefore, connectivity between these networks may reflect purposeful motor performance.

In conclusion, altered resting-state connectivity was found for all HMD in multiple, sometimes overlapping, brain regions, and clinical measures correlated with altered connectivity. This underscores the growing notion that HMD should not be considered the result of disruption of one brain region, but should instead be viewed as network disorders. Nevertheless, some crucial nodes play an important part in these networks, such as the striatum in chorea, and the cerebellum in myoclonus and tremor. Furthermore, these findings in specific brain regions help to elucidate the pathophysiology underlying several HMD. Additionally, given that rs-fMRI is sensitive to differences in clinical characteristics, rs-fMRI may be a promising biomarker to track progression, severity, and treatment effects of HMD. Importantly, it should be noted that the results were often in opposite directions, which could be due to a complex interplay of factors, including differences in methodologies used and how brain regions interact. Therefore, novel methodologies are necessary that can better capture neural networks underlying brain function, also taking into account the time-varying aspect of network connectivity.

## Funding

R.S.M. received funding from the Junior Scientific Masterclass from the University of Groningen, University Medical Center Groningen. M.A.J.T. reports grants from the Dutch Organization for Health Research and Development ZonMW Topsubsidie (91218013), the European Fund for Regional Development from the European Union (01492947) and the province of Friesland, from the Dystonia Medical Research Foundation, from Stichting Wetenschapsfonds Dystonie, from Fonds Psychische Gezondheid, from Phelps Stichting, and an unrestricted grants from Actelion and AOP Orphan Pharmaceuticals AGAOP Orphan Pharmaceuticals AG. The author(s) confirm(s) independence from the sponsors; the content of the article has not been influenced by the sponsors.

Several authors of this publication are members of the European Reference Network for Rare Neurological Diseases - Project ID No 739510.

## CRediT authorship contribution statement

**Ramesh S. Marapin:** Conceptualization, Methodology, Formal analysis, Visualization, Data curation, Writing – original draft. **Harm J. van der Horn:** Conceptualization, Methodology, Visualization, Writing – review & editing, Supervision. **A.M. Madelein van der Stouwe:** Conceptualization, Methodology, Writing – review & editing, Supervision. **Jelle R. Dalenberg:** Methodology, Visualization, Writing – review & editing. **Bauke M. de Jong:** Conceptualization, Writing – review & editing. **Marina A.J. Tijssen:** Conceptualization, Writing – review & editing, Supervision.

## Declaration of Competing Interest

The authors declare that they have no known competing financial interests or personal relationships that could have appeared to influence the work reported in this paper.

## Data Availability

Data will be made available on request.
