# Supplementary material for: Altered brain connectivity in hyperkinetic movement disorders: A review of resting-state fMRI
Source: Neuroimage Clin. 2022 Dec 24;37:103302. doi: 10.1016/j.nicl.2022.103302 (PMC9868884; doi:10.1016/j.nicl.2022.103302)
Supplement: Supplementary data 1 [file mmc1.docx]

**Identification of studies via other methods**

**Identification of studies via databases and registers**

Records removed *before screening*:

Duplicate records removed
(n = 29)

Records identified from:

Citation searching (n = 10)

etc.

Records identified from*:

Databases (n = 1052)

**Identification**

Records excluded

(n = 818)

Records screened

(n = 1023)

Reports not retrieved

(n = 0)

Reports not retrieved

(n = 0)

Reports sought for retrieval

(n = 10)

Reports sought for retrieval

(n = 205)

**Screening**

Reports excluded: 57

Wrong patient group (n = 24)

Small sample size (n = 12)

Analysis not of interest (n = 10)

Already analyzed (n = 9)

No resting-state fMRI (n = 1)

Review & case report (n = 1)

Reports assessed for eligibility

(n = 10)

Reports excluded
(n = 0)

Reports assessed for eligibility

(n = 205)

Studies included in review

(n = 158)

Reports of included studies

(n = 158)

**Included**

**Supplementary Figure 1.** Flowchart of included and excluded studies.
