# Supplementary material for: Altered brain connectivity in hyperkinetic movement disorders: A review of resting-state fMRI
Source: Neuroimage Clin. 2022 Dec 24;37:103302. doi: 10.1016/j.nicl.2022.103302 (PMC9868884; doi:10.1016/j.nicl.2022.103302)
Supplement: Supplementary data 2 [file mmc2.docx]

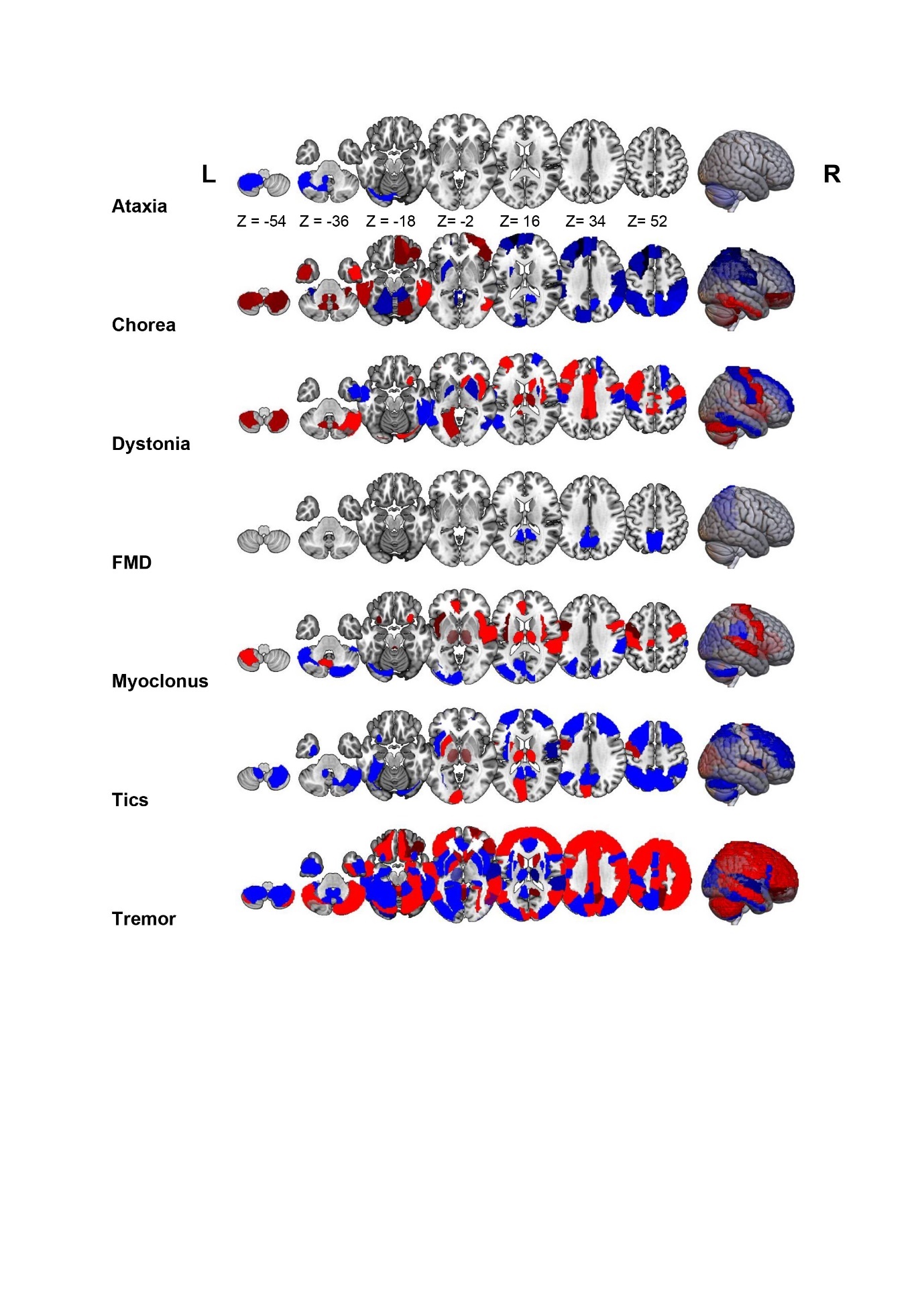


**Supplementary Figure 2**. Brain regions that showed significant differences in resting-state connectivity between patients and healthy participants, using ALFF and ReHo analysis across several HMD. Blue and red values indicate reduced and increased regional connectivity, respectively, where brighter values correspond to higher effect sizes according to the standardized mean difference.


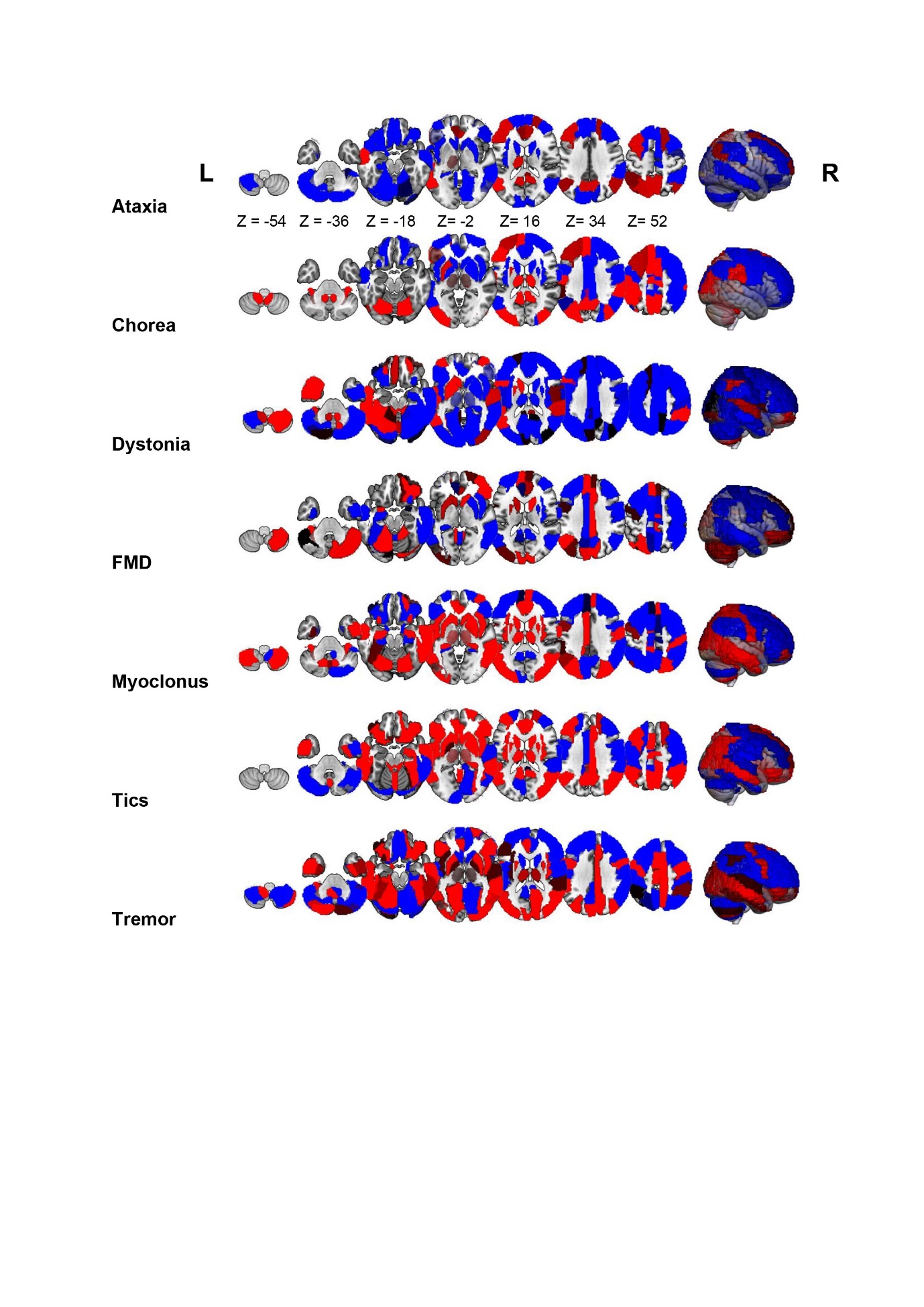


**Supplementary Figure 3**. Brain regions that showed significant differences in resting-state connectivity between patients and healthy participants, using FC and graph analysis methods across several HMD. Blue and red values indicate reduced and increased regional connectivity, respectively, where brighter values correspond to higher effect sizes according to the standardized mean difference.


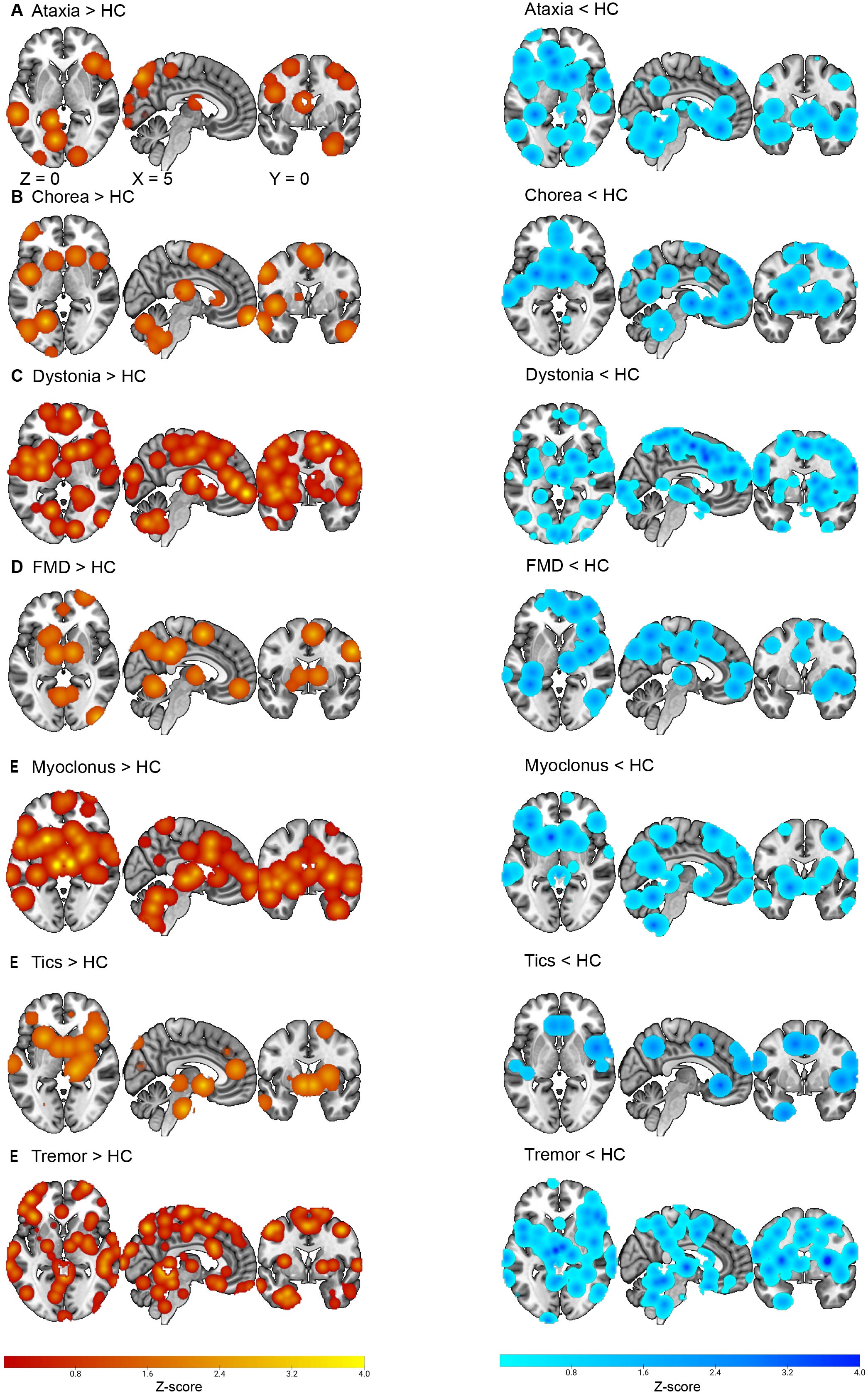


**Supplementary Figure 4.** Anatomical location of significant findings for each phenotype. Unthresholded z-score maps are shown for hyperconnectivity (red) and hypoconnectivity (blue) in patients.
