# Supplementary material for: Altered brain connectivity in hyperkinetic movement disorders: A review of resting-state fMRI
Source: Neuroimage Clin. 2022 Dec 24;37:103302. doi: 10.1016/j.nicl.2022.103302 (PMC9868884; doi:10.1016/j.nicl.2022.103302)
Supplement: Supplementary data 3 [file mmc3.docx]

# **Supplementary Material**

## S1. Search terms used for the literature search using Pubmed.

The following search terms were used for the literature search using Pubmed. Additionally, we performed a targeted search using articles’ reference lists to identify additional studies of interest.

("Magnetic Resonance Imaging"[Mesh] OR mri*[tiab] OR magnetic resonance imag*[tiab] OR fmri*[tiab])

AND

("Movement Disorders"[Mesh] OR “Dyskinesias”[Mesh] OR "Tremor"[Mesh] OR "Ataxia"[Mesh] OR "Myoclonus"[Mesh] OR "Dystonia"[Mesh] OR "Dystonic Disorders"[Mesh] OR "Chorea"[Mesh] OR "Tics"[Mesh] OR movement disorder*[tiab] OR dyskine*[tiab] OR hyperkine*[tiab] OR tremor*[tiab] OR ataxia[tiab] OR myoclon*[tiab] OR dystoni*[tiab] OR chorea*[tiab] OR tic[tiab] OR tics[tiab] OR tic disorder*[tiab] OR conversion disorder*[tiab] OR functional neurological disorder*[tiab])

AND

("Rest"[Mesh] OR resting state[tiab] OR rest[tiab])

## S2. Inclusion criteria and screening procedure

Three of the authors (RM, AMMS, and HJvdH) independently reviewed the titles and abstracts, blinded to authors and journal titles, using an Excel workbook designed specifically for screening.^1^ This Excel workbook was used to manage the records and data throughout the review. Items were reviewed in pairs, and in cases where there was disagreement, cases were discussed by the two article reviewers until consensus was reached. If consensus could not be reached, the third reviewer provided final arbitration. We included studies if they reported resting-state fMRI findings in patients with the HMD and FMD described above. Studies were excluded if they did not perform an analysis considered of interest (e.g., solely focusing on cognitive rather motor symptoms). Only articles published in English and available online were included. Human subjects of any age were included. We set the minimum number for patients investigated at 10. We did not set a limit on the year that the studies were published.

Prior to screening all titles and abstracts, the article reviewers reviewed the first 10 articles together in order to reduce the interrater variability. Afterwards, we calculated Cohen’s kappa statistic to test interrater reliability, which reflects the extent of agreement among the article reviewers.^2^ We did this to ensure that all reviewers fully understood the aims and eligibility criteria of the systematic review and thus further reducing the interrater variability. To test the interrater reliability, we used an Excel workbook designed specifically for the Cohen’s kappa interrater reliability test.^1^ Only titles and abstracts were reviewed; authors and journal titles were not included in the sample. After independent review, a Cohen’s kappa of 0.85 was reached, which is considered a strong level of agreement.^2^

## S3. Extracted information

We extracted the following data from the studies that were included: the HMD phenotype, the numbers of participants, the type of analysis conducted (e.g., functional connectivity analysis), clinical measures of interest included in potential correlational analyses, and the results of the main analysis. We primarily focused on clinical measures with a clear relation to motor functioning, such as the age of onset, disease duration, and motor severity, and excluded cognitive and psychometric measures. To evaluate the consistency and specificity of findings across different studies, we also extracted t, z, and/or p values from each study, when available. These values were then converted into the standardized mean difference (SMD) between patients and healthy participants using Wilson’s web-based effect-size calculator to make them comparable across studies.^3^

## S4. Reporting of results

The main methods used by the studies included in this review were: amplitude of low frequency fluctuations, regional homogeneity, graph-based network analysis, and functional connectivity analysis). To report statistical findings, we calculated an SMD for each study result when possible. For ALFF and ReHo, this entailed assigning a SMD for each affected brain region. For functional connectivity analyses describing impaired correlations involving multiple brain regions, each involved brain region was assigned a SMD according to the strength and direction of the impaired correlation, e.g., a positive SMD for brain regions showing increased connectivity with other regions. In case multiple t, z, and/or p values were reported within the same AAL brain region, the largest value was chosen to calculate the SMD. Results in opposite directions within the same study and AAL brain region were regarded as missing values for the SMD. In case there were only P-values reported, SMD was given a positive or negative value based on the direction of the effects reported by the authors in the main text.

To summarize findings per HMD phenotype, we used the SMD in the following ways. First, we calculated the absolute value (i.e., only positive values) of the SMD, in order to reflect the degree of involvement of each region per HMD phenotype (Figs. 1-2). Furthermore, we constructed HMD brain signature maps by averaging SMDs across studies and AAL brain regions, to describe the direction (i.e., positive or negative) of our findings (Supp. Figs. 2-3). It should be noted that several brain regions showed inconsistent results, i.e., regions exhibiting both increased and decreased connectivity within the same HMD phenotype. Therefore, caution is warranted when interpreting these results.

The activation likelihood estimation (ALE) approach was used to provide a quantitative synthesis of the rs-fMRI studies included in this review.^4^ This approach evaluates brain regions in which the convergence of reported hyperconnectivity or hypoconnectivity across studies is higher than would be expected by chance. This analysis was done using the GingerALE software (version 3.0.2; <http://www.brainmap.org/ale/>). With ALE, there is an important consideration that the spatial coordinates referring to significant findings (foci) in each study are associated with some degree of spatial convergence. Significant results of studies are therefore not treated as exact points, but rather as centers of a tridimensional Gaussian probability distribution, with the center indicating the highest probability of activation.^5^ ALE analysis involves modelling these foci as probability distributions whose width is based on empirical estimates of the spatial uncertainty due to the between-subject and between-template variability underlying neuroimaging data. ALE results are subsequently assessed against a null-distribution of random spatial association between experiments, resulting in random-effects inference.^4^ For the complete description and procedure of the ALE method, we further refer the reader to the Eickhoff et al. (2012) and the GingerALE manual, which can be found on the website: <http://www.brainmap.org/ale/>

## S5. Visualization of results

For the changes in resting-state signal fluctuations and synchronization, results for all HMD were visualized by weighing each brain region in the AAL atlas according to the degree of involvement (Figs. 1-2) and direction of connectivity (Supp. Figs. 2-3) and overlaying this on a volume rendered brain in Montreal Neurological Institute (MNI) space, using MRIcron and MRIcroGL (Figs. 1-2 and Supp. Figs 2-3).^6^ Functional connectivity and graph analysis assesses the brain as an integrated network and focuses on the connectivity between different brain areas, whereas ALFF and ReHo analyses focus on regional brain connectivity.^7^ As these methods measure different properties of brain functioning, we visualize the results of these methods separately.

Furthermore, we also conducted hierarchical cluster analysis (HCA) to provide additional insights into commonalities and differences across HMD phenotypes.^8^ Essentially, HCA attempts to group objects (i.e., HMD phenotypes) with similar features into clusters. The results of our HCA were then visualized in the form of a heat map, which is a graphical data representation where the individual values contained in a numerical matrix are represented as colors (Fig. 4).^9^ For visibility purposes, in this figure we grouped the AAL regions used in this study into larger scale networks, based on a previous study.^10^

1. VonVille, H. Excel Workbooks and User Guides for Systematic Reviews. https://www.yopl.info/post/excel-workbooks-and-user-guides-for-systematic-reviews (2021).

2. McHugh, M. L. Interrater reliability: The kappa statistic. *Biochem. Medica* **22**, 276–282 (2012).

3. Wilson, D. B. Practical Meta-Analysis Effect Size Calculator [Online calculator]. https://www.campbellcollaboration.org/research-resources/effect-size-calculator.html.

4. Eickhoff, S. B., Bzdok, D., Laird, A. R., Kurth, F. & Fox, P. T. Activation likelihood estimation meta-analysis revisited. *Neuroimage* **59**, 2349–2361 (2012).

5. Cortese, S., Aoki, Y. Y., Itahashi, T., Castellanos, F. X. & Eickhoff, S. B. Systematic Review and Meta-analysis: Resting-State Functional Magnetic Resonance Imaging Studies of Attention-Deficit/Hyperactivity Disorder. *J. Am. Acad. Child Adolesc. Psychiatry* **60**, 61–75 (2021).

6. Rorden, C. & Brett, M. Stereotaxic display of brain lesions. *Behav. Neurol.* **12**, 191–200 (2000).

7. Lv, H. *et al.* Resting-state functional MRI: Everything that nonexperts have always wanted to know. *Am. J. Neuroradiol.* **39**, 1390–1399 (2018).

8. Zhang, Z., Murtagh, F., Poucke, S. Van, Lin, S. & Lan, P. Hierarchical cluster analysis in clinical research with heterogeneous study population: highlighting its visualization with R. *Ann. Transl. Med.* **5**, (2017).

9. Toddenroth, D., Ganslandt, T., Castellanos, I., Prokosch, H. U. & Bürkle, T. Employing heat maps to mine associations in structured routine care data. *Artif. Intell. Med.* **60**, 79–88 (2014).

10. Allen, E. A. *et al.* Tracking whole-brain connectivity dynamics in the resting state. *Cereb. Cortex* **24**, 663–676 (2014).
